# A Systematic Review on the Continuous Cropping Obstacles and Control Strategies in Medicinal Plants

**DOI:** 10.3390/ijms241512470

**Published:** 2023-08-05

**Authors:** Muhammad Zeeshan Ul Haq, Jing Yu, Guanglong Yao, Huageng Yang, Hafiza Amina Iqbal, Hassam Tahir, Hongguang Cui, Ya Liu, Yougen Wu

**Affiliations:** Sanya Nanfan Research Institute of Hainan University, School of Tropical Agriculture and Forestry, Hainan University, Sanya 572025, China

**Keywords:** medicinal plants, continuous cropping obstacles, microbial community, molecular mechanisms, control strategies

## Abstract

Continuous cropping (CC) is a common practice in agriculture, and usually causes serious economic losses due to soil degeneration, decreased crop yield and quality, and increased disease incidence, especially in medicinal plants. Continuous cropping obstacles (CCOs) are mainly due to changes in soil microbial communities, nutrient availability, and allelopathic effects. Recently, progressive studies have illustrated the molecular mechanisms of CCOs, and valid strategies to overcome them. Transcriptomic and metabolomics analyses revealed that identified DEGs (differently expressed genes) and metabolites involved in the response to CCOs are involved in various biological processes, including photosynthesis, carbon metabolism, secondary metabolite biosynthesis, and bioactive compounds. Soil improvement is an effective strategy to overcome this problem. Soil amendments can improve the microbial community by increasing the abundance of beneficial microorganisms, soil fertility, and nutrient availability. In this review, we sum up the recent status of the research on CCOs in medicinal plants, the combination of transcriptomic and metabolomics studies, and related control strategies, including uses of soil amendments, crop rotation, and intercropping. Finally, we propose future research trends for understanding CCOs, and strategies to overcome these obstacles and promote sustainable agriculture practices in medicinal plants.

## 1. Introduction

Continuous cropping obstacles (CCOs) cause crop failure, root rot disease, yield decline, poor quality, replantation, soil sickness, and even the death of seedlings in the same plot of the same plant [1,2]. The continuous cropping (CC) efficiency of medicinal plants may be affected by numerous issues, including soil nutrient deficits, a rise in the allelopathic autotoxicity of root exudates, the buildup of fungal pathogens, and imbalances in the soil microbial community [3,4]. CC infestation can be extremely detrimental to ginseng plants, resulting in decreases in yields of up to 80–100%, severe root rot illnesses, the falling off of fibrous roots, and, in some cases, plant death [5,6]. Similarly, in the plant *Fagopyrum tataricum*, yield declined up to 6.36% in the first year, 24.85% in the second year, 78.62% in the third year, and 83.10% in the fourth year of CC, which caused severe economic losses. The availability of nutrients, soluble proteins, soil enzyme activities, total chlorophyll contents, and the number of actinomycetes in leaves gradually decrease during CC [1]. In a previous study, CC ginseng seedling survival rate dropped to 30%, and 70% of the plant was burnt off and overrun with scars on the plant’s skin and its roots. In continuous cropping areas, root rot occurred, on average, 23.9% of the time, which is 3.5 times more often than on newly cultivated ground [7]. The productivity and quality of curative plants, including *Lepidium meyeni*, *Panax ginseng*, *Panax notoginseng*, *Andrographis paniculate*, and *Rehmannia glutinosa*, were significantly impacted by CCOs (Table 1) [8,9,10].

In summary, by inhibiting plant defense enzymes, CCOs upset the equilibrium of the relative oxygen species (ROS) metabolism, allowing ROS to build up in overabundance, ultimately resulting in membrane lipid peroxidation. There were notable differences in levels of degraded aromatic compounds, microbiome activity, chitin degradation, nitrogen fixation, and atrazine metabolism. Soil degradation and the microbial inhabitants’ edifice imbalance were the primary causes of CCOs [11]. Transcriptomic research displayed significant variations in the soil’s microbiota during CC [12]. *Rehmannia glutinosa* L. plants suffering from CCOs had small leaves, losses in root ATPase activity, slow growth rates, and decreased root activity [13]. Negative microbe–plant interactions were shown to be mostly caused by structural changes in the microbial population, in addition to soil fertility decline, soil nutrient imbalance, and rhizospheric soil autotoxin buildup [14,15]. CC substantially corresponds with a rise in the richness of the soil-borne fungus *Fusarium oxysporum* by reducing the abundances of *Bacillus, Rhizobacteria*, and *Pseudomonas*, all of which promote plant development [16,17]. It is estimated that it takes from 4 to 10 years for medicinal plants and planted soils to naturally recover after CC [18]. Farmers use a variety of artificial agricultural management techniques, including crop rotation, soil sterilization, fertilizer application, and fumigation, to speed up the recovery process and prepare these soils for continuous ginseng farming [19,20]. However, the widespread use of agrochemicals in ginseng raises questions about the safety of both humans and the environment. A different approach to removing ongoing cropping difficulties and reducing cropping fields’ recovery period may lie in using eco-friendly bio-fertilizers [21]. Different medicinal plants’ adaptive reactions in response to CCOs are shown in Table 1.

In this review, we have outlined the problem of CC in medicinal plants. In order to develop medicinal plant resources more wisely, we also identified the current issues and proposed some appropriate remedies. Identifying the response of various medicinal plant species and CC on genome-wide transcriptome profiles, the structure and composition of soil microbial communities, candidate genes, and pathways related to CC response, intercropping, soil amendments, crop rotation, and understanding related molecular mechanisms are the goals of this study. In order to cope with CC soil, comprehensive procedures are now being used, including physical, chemical, and agronomic measures; positive results have been obtained in reducing the impact of continuous cropping challenges. Finally, potential research directions for the future are also discussed.

**Table 1:** Observed adaptive responses to continuous cropping obstacles of different medicinal plants.

Plant Name	Plant Adaptive Reaction	Reference
*Pinellia ternata*	Plant growth decline, reductions in photosynthetic pigments, soluble sugar, yield, and alkaloids	[22]
*Panax ginseng*	Heavy metal and allelochemicals’ accumulation, imbalance in the rhizosphere micro-ecosystem, deterioration of physical and chemical properties, and soil-borne disease	[8]
*Lilium lancifolium*	Accumulation of pathogenic bacteria in the rhizosphere, causing severe root rot disease	[23]
*Lepidium meyeni*	Reduced N, P, K contents, soil organic matter, dry and fresh weight	[9]
*Andrographis paniculata*	Increased bacterial diversity, Acidobacteria and Zygomycota phyla and fungal genus *Mortierella*, alongside decreased fungal diversity and bacterial genus *Pseudolabrys*	[10]
*Rehmannia glutinosa* L.	Reduction in root activity, chlorophyll contents, and leaf size, and loss of ATPase activity in roots, growth rate, and photosynthetic efficiency	[13,24]
*Coffea arabica* L.	Increased major soil-borne diseases and yield decline	[25]
*Angelica sinensis* (Oliv.) Diels	Increased SS, EL, proline and MDA, alongside decreased CAT, SOD, POD, yield, chlorophyll contents, plant growth, photosynthetic rate, and essential oil contents	[26]
*Panax notoginseng* (Burk.)	Decreased production and tuber quality, and increased seedling mortality rate	[27]
*Pogostemon cablin* (Benth.)	Oxidative stress, nutritional deficiency, core metabolic disorder, and genotoxicity	[28]
*Codonopsis tangshen* (Oliv.)	Increased total soluble protein, MDA, and CAT activity, alongside reduced SOD, Chl. a (chlorophyll a), Chl. b (chlorophyll b), and total Chl. (total chlorophyll)	[29]
*Panax Ginseng* C.A. Mayer	Yellow spot disease on leaves and stems, and serious fibrous abscission in roots (rust rot and root rot)	[30]
*Salvia miltiorrhiza* Bge.	Loss of fresh weight, lipophilic, lithospermic acid, root length and diameter, salvianolic acid B, hydrophilic contents, and significant yield decline	[31]
*Coptis chinensis* Franch.	Decreased Proteobacteria and alpha diversity, increased Acedobacteria, change in rhizosphere species, and pH slightly acidic	[32]
*Radix pseudostellariae* L.	Reduction in acid protease and chitinase, alongside increased sucrose, urease, protocatechuic acid, and cellulose	[33]
*Panax quinquefolium*	Reduced soil pH, alpha fungal diversity, and bacterial diversity	[34]
*Panax quinquefolius* L.	Increased NO3-N, soil salt, and NH4+-N, alongside decreased cellulose activity, soil pH, alkaline phosphate, and C: N ratio	[21]

## 2. Methods

**Figure 1:**
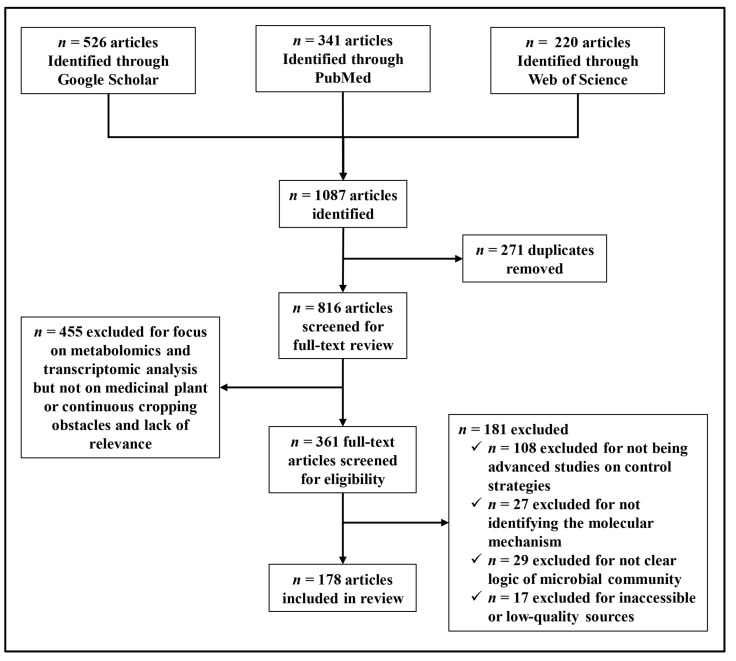
Continuous cropping obstacles in medicinal plants flow diagram, demonstrating search pathway results and included articles.

We conducted a systematic review of Google Scholar, Web of Science, and Pub-Med/continuous cropping and medicinal plants libraries using varied keywords: “medicinal plants”, “continuous cropping obstacles”, “microbial community”, “molecular mechanisms”, “transcriptomic analysis”, “metabolomics study”, “gene regulation”, “control strategies”, “soil amendments”, “crop rotation”, and “intercropping”, in numerous amalgamations. We evaluated the facts from research articles as well as review articles to create a systematic review of continuous cropping obstacles in medicinal plants within recent publications. This study identified 178 advanced articles for inclusion (Figure 1).

The results and discussion were analyzed and arranged according to the principal relationships: “causes of continuous cropping obstacles”, “molecular mechanisms in continuous cropping obstacles”, and “medicinal plants and control strategies”, with a special emphasis on transcriptomic and metabolomics studies. Finally, we recommended future study directions regarding control strategies in the field of continuous cropping in medicinal plants.

## 3. Results and Discussion

### 3.1. Causes of Continuous Cropping Obstacles

#### 3.1.1. Soil Microbial Community

CCOs are often caused by a mix of abiotic and biotic conditions, containing decreasing soil physicochemical properties, plant autotoxicity, and changes in the composition and diversity of microbial communities [35]. Microbial community imbalance in American ginseng reduces the metabolic function of the plant in a continuous cropping system, decreases bacterial community, and increases fungal diversity, which affects soil health and quality [36,37]. Medicinal plant development and growth, plant robustness against environmental stress, and plant nutrient absorption may all be influenced positively by rhizospheric bacteria [38]. The most abundant and diverse microflora in each gram of soil are bacteria, which are estimated to include millions of bacterial species and billions of cells [39]. The bacterial population of soil is very important in the development of plant disease [40]. However, different root exudates secreted by different plant species can change the soil bacterial community’s makeup, and changing cultivation methods may also change the microbial communities [41]. Rhizosphere soil has considerable accumulations of physicochemical traits, and the prevalence of important microbes and certain phenolic acids caused illness in a CC system in 12 years of strawberry crop study [42].

CCOs are created and reduced by the soil microbial community due to their strong connection with the absorption of the plant’s nutrients, infectious diseases, and the immune system [43]. During CC, wilt disease may be prevented by bacteria which are locally root-associated [44]. Moreover, plants enlist bacteria to aid in nutrient uptake and resist diseases and stress [45]. However, in a single cropping system, diversity declines among ammonia oxidizers and functional groups with the passage of time, while ammonia oxidation genes remain concentrated. This causes ammonia to oxidize quickly into nitrate, which causes nitrogen loss and poor nitrogen nutritional efficiency [46]. CC depletes the variety of soil bacteria and the relative abundance of beneficial microorganisms in plants while increasing the amount of pathogenic (*Ralstonia*) bacteria, which causes an epidemic of bacterial wilt disease [47]. The soil–plant microbiome has great significance in plant production control, which is essential to investigate the microbial community of plants in the field of CC [48]. When anaerobic soil disinfestation was used to address CCOs, soil nutrient availability modifications connected with soil microbial population changes over the passage of time [49]. In contrast, PGRP (plant growth-promoting rhizobacteria) improved the soil-borne pathogen immunity of plants, and raised output in CC soil, tending to reduce soil-borne illness [50]. Several molecular analytical techniques, like fluorescence in situ hybridization, 454 pyrosequencing analysis, fragrant length polymorphism terminal restriction, high-throughput sequencing, and denaturing gradient gel electrophoresis, are applied to analyzing a variety of microorganisms in rhizosphere soil, along with evolution and relative abundance in CC systems [51,52]. Preserved functional contribution characteristics and 16S rRNA homology-based PICRUSt (phylogenetic investigation of communities by reconstruction of unobserved states) were utilized to foresee bacterial community functional structures [53].

#### 3.1.2. Nutrient Availability

##### Soil Organic Matter and Organic Carbon Contents

The main factors and markers of soil’s strength are its carbon contents and organic matter, which are necessary for the fertility, quality, functionality, and sustainability of soil [54]. The growing body of research suggests that CC combined with damaging tillage techniques may have a deleterious impact on soil organic matter in agriculture [55]. In contrast to the soil that has constantly been planted with maize, fallow land has considerably greater soil organic C (soil organic carbon) contents [56]. The two major classes of soil organic C (aromatic C and O-alkyl)’s relative composition, through the soil depth profile, are altered by a continuous cropping system as opposed to a rotational one [57]. In various experiments, the effects of cereal monoculture cropping systems and rotations have been found to be dominated by leys on the levels of soil organic C [58]. Ley-dominated rotations significantly increased soil organic C levels compared to cropping systems of cereal monoculture. The soil under continuous cotton has a substantially lower soil C concentration than soil under continuous sorghum and maize cultivation [59,60]. Cotton crop residues and toxic metabolites may inhibit the halting of crop litter, and hence may limit the quantity of soil organic C, which may be to blame for decreased C concentrations in soil under cotton, as opposed to other crops. In the CC of sesame, a decrease in the soil organic C level was also documented [61]. Due to conflicting data from various soil and crop systems, it may be challenging to understand how soil organic C and matter are affected by continuous cropping. However, under CC systems that use conservation-oriented practices (e.g., manure input and reduced tillage), soil organic matter and C levels may remain the same, or even rise [62]. To establish the effects of CC on soil organic matter levels, several crops and soil parameters (soil biodiversity, tillage intensity, plant-derived metabolites, use of fertilizer, crop root systems, and the input of crop residue) may be relevant [63].

##### Carbon (C) and Nitrogen (N) Cycling and Mineralization

One important sign of soil fertility and the health of agroecosystems is soil carbon and nitrogen mineralization [64]. Soil structure, the texture of the soil (clay vs. sandy), crop residue amount, and fertilizer application among other factors influence the soil C content and N mineralization damage caused by CC [65]. Clay soil is more adroit than sandy soil in organic and inorganic substance binding. Additionally, nutrient mineralization is unpredictable; growers misuse N fertilizers, which could affect N mineralization and cause losses in soil subjected to CC [66]. In corn CC systems, usage of fertilizer (NH^+^_4_-N) reduced organic matter mineralization (gross ammonification declined from 13% to 21%), limiting the nitrogen cycle and altering soil fertility [67]. Furthermore, a higher C and N ratio in a few crops (maize instead of soybean) wastes may elucidate differences in carbon and nitrogen mineralization beneath the CC [68]. Meanwhile, increased N and stronger microbial activity are predicted to result in increased nutrient levels and C mineralization in the soil under legume crops like soybean, under cropping systems of continuous corn and corn–soybean. Additionally, soil temperature reduction and an increase in maize covering (rather than soybean residues) may have an impact on nutrient mineralization [69]. Unfertilized continuous corn soils have less N mineralization than soybean–corn cropping, while several other studies have found less N mineralization in continuous corn than in corn–soybean cycle cropping [70]. Mineralization of carbon and nitrogen was enhanced more by fertilized soil under a corn CC system than unfertilized control soil [71]. Because CC systems are less capable of sequestering nutrients, which might impact the soil ecosystem nutrient cycle, higher nutrient deficiencies, primarily through leaching or surface run, are anticipated [72]. Furthermore, monoculture CC systems result in substantially fewer diversified crop residues, which has an impact not only on the movement of nutrients but also on the physical and biological soil ecosystem mechanisms that control nutrient cycling and mineralization [73]. To forecast how CC will affect soil health and productivity, it is crucial to have a thorough grasp of how CC affects nitrogen cycling.

##### Nutrient Deficiencies

Nitrogen-rich fertilizer usage, the intensity of tillage, and the breakdown of the crop residues (and therefore, amplified organic acid formation in soil) are primarily responsible for the decline in soil pH in the CC system [65]. The availability of vital macro and micronutrients (nitrogen, potassium, phosphorous, magnesium, calcium, and molybdenum) is significantly impacted by soil acidity, in addition to the detrimental effects it has on soil organisms, as previously described [39]. The deleterious effect of soil acidity on agricultural crop yields has been stated in numerous studies [74]. Furthermore, CC undermines soil resources, which may deplete important minerals and tip the balance of nutrients in the soil. In US corn–soybean belt zones in three northern states, continuous corn revealed considerably lower soil potassium levels than a crop rotational system in soil [55]. The soil beneath American ginseng had micronutrient levels of Fe (Iron) (80%), Mn (Manganese) (113%) and Cu (Copper) (99%) that were higher than those in maize CC soil [75]. Additionally, CC under some conventional techniques provides unpleasant circumstances that prevent plants from absorbing enough nutrients from the soil, which may also have an adverse effect on plant development and crop output [76]. Although there are multiple cases of soil nutrient deficits under CC, salt levels and the cation exchange capacity in soil are also affected by these practices, leading to soil deterioration. Furthermore, soil composition, bulk and density, aeration, lowering water infiltration, and nutrient movement are all increased by the CC system, and these are key markers of soil health [77]. Lastly, amongst other things, CC may alter the variety of modifications that regulate the physiochemical environment of the soil, leading to the buildup of harmful compounds and nutrient leaching, which degrade agricultural water and soil quality over time [78].

##### Physiochemical Properties of Soil

The following soil physiochemical characteristics (moisture, water holding capacity, pH, nutrient content, conductivity, capacity for cation exchange, alkalinity, compaction, soil bulk density, temperature, microclimate, acidity, saturation percentage, buffering capacity, porosity, etc.) are not exhaustive. Numerous soil physicochemical characteristics, including pH and concentrations of vital micronutrients and macronutrients, might be impacted by CC [39]. Soil pH has a greater impact on archaeal and bacterial community composition than the composition of fungi [79]. Similarly, researchers have also reported that the microbial community in CCOs is also affected by changes in soil pH [27]. Additionally, soil moisture, N, and P contents also directly or indirectly influence the soil microbial community’s spatial arrangement [80]. The two most crucial aspects of soil are its pH and CEC (cation exchange capacity), which control the biochemistry and biology of the soil, as well as plant development. However, continuous cropping affects the physiochemical qualities of the soil, and is influenced by meteorological and soil conditions. In semi-arid and dry agroecosystems, CC raises the pH of the soil by enhancing sodicity, salinity, and alkalinity, which might impact the accessibility of the nutrients in soil to plants [81].

#### 3.1.3. Allelopathic Effects

Autotoxicity, a specific kind of allelopathy, is a phenomenon wherein the same plant’s development and growth are prevented by allelochemicals [82]. The primary mechanisms through which it inhibits plant growth are exchanging cells’ ultra and microstructures, affecting respiration and photosynthesis, disrupting the antioxidant system, inhibiting water and nutrient uptake, increasing cell membrane permeability, inhibiting cell division and elongation, interfering with the growth regulator system, and affecting nucleic acid and protein synthesis and metabolism [83]. Several scientists have extracted and identified allelochemicals from various plants in recent years, primarily from monoterpenes, organic acids, flavonoids, phenolic acids, terpenoids, plant volatiles, and coumarins [84]. Phenolic acids, being the most extensively researched and actively studied compounds, have emerged as a central focus in allelopathy research concerning soil diseases [85]. Numerous studies have revealed that phenomena such as allelopathy or autotoxicity might have a negative impact on continuously grown crops [86]. Phenolic acids, produced during plant growth as the primary allelochemicals, represent the principal CC barriers for numerous terrestrial plants. Many agricultural and medicinal plants have been documented to be negatively impacted by phenolic acids [87]. Notably, nine phenolic acids (vanillic acid, vanillin, salicylic acid, protocatechuic acid, syringic acid, benzoic acid, coumaric acid, p-hydroxybenzoic acid and phthalic acid), together with ferulic acid and cinnamic acid have been discovered to hinder ginseng radicle growth. This inhibiting effect was elevated with the increasing concentration of phenolic acids, indicating phenolic acids had a direct obstructive impact on ginseng growth. Nevertheless, there are other microbes that communicate with the soil phenolic acids, particularly certain probiotics (*Burkholderia* and *Spingomonas*) and diseases that affect agriculture [88]. Ferulic acid stimulated *Fusarium oxysporum* development at low doses, but reduced it at high doses [89]. A high quantity of phenolic acids can prevent the growth of harmful bacteria, therefore enhancing ginseng growth. On one hand, phenolic acids can be utilized as substrates to boost helpful microbes. As a result, increased phenolic acid concentrations can both hinder ginseng growth as allelochemicals and enhance plant growth by promoting salutary microbes and inhibiting non-beneficial microorganisms. Allelochemicals’ indirect and direct activities conflict with plant growth responses, much as the allelopathic response of many plants to the invasive species *Phytolacca americana* [90].

#### 3.1.4. Others

Changes in soil pH have a significant impact on the composition of the soil microbial community, which might cause problems for ongoing crop growth [91]. Numerous other elements, such as soil moisture, nitrogen, and phosphorus content, may also have an indirect or direct impact on spatial soil microbial community structure [80]. A significant element that influences the variety and structure of the soil microbial community is the nitrogen concentration in CC [92]. According to an earlier research, *P. ternata* seedling development in CCOs is significantly inhibited by substances like chlorogenic acid, vanillin, benzofuran, vanillic acid, syringic acid, ferulic acid, protocatechuic acid, gallic acid, syringaldehyde, etc. As a result, these substances are regarded as primary allelochemicals and autotoxins of *P. ternata* [93]. Under CCO, some plants’ rhizospheres accumulate phenolic acid, which affects the soil microbial ecology [94]. Soil microbial community resistance is also decreased by CC, and as years pass, the bacterial community’s fungal diversity can change, pathogen antagonistic bacteria, which are the main cause of CCOs, can be abridged [95]. In addition, a number of studies have demonstrated that similar cultivars have distinct nutrient demand types and soil absorption ratios, which lead to soil nutrient imbalance due to CC [96]. Current research suggests that CC causes an imbalance in the diversity and structure of endophytic and rhizospheric soil fungal communities, as well as the rapid accumulation of fungal pathogens [97]. Therefore, a stable and vigorous community of rhizospheric, fungal and endophytic bacteria is crucial for maintaining long-term continuous cropping and a steady crop yield.

### 3.2. Molecular Mechanisms

#### 3.2.1. Transcriptomic Analysis

**Figure 2:**
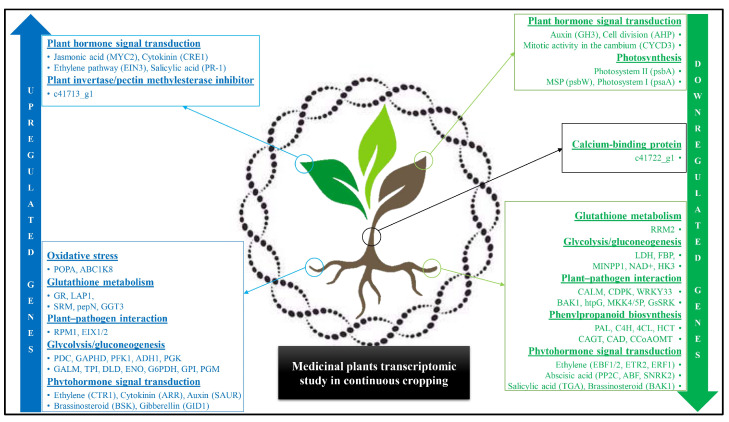
Different medicinal plants’ transcriptomic studies with continuous cropping obstacles. The blue color shows upregulated genes, while the green color shows downregulated genes.

Transcriptomic analysis is a cutting-edge biologic discipline which utilizes high throughput, high resolution, and sensitive methods to analyze both model and non-model entities. Transcriptomic studies can assist researchers in assessing medicinal plants’ controlling mechanisms and functional genes to improve cultivation systems and breeding methods in CC [98]. Additionally, crucial transcriptional components have been found in the ginseng (*Panax ginseng*) plant’s transcriptional response to benzoic acid (Autotoxin), a soil root exudate compound that is continually farmed [99]. Recently, proteome modification of leaves with regard to CC in the patchouli (*Pogostemon cablin*) plant has demonstrated how CC changed the protein expression involved in the metabolism of amino acids, energy, and carbohydrates [100]. The 762 DEGs discovered via transcriptome profiling contain upregulated and downregulated genes (430 and 332, respectively), due to CC in the *Codonopsis tangshen* plant [101]. Additionally, a pathway enrichment analysis showed that CC-upregulated genes are involved in tyrosine catabolism and phenylalanine, tyrosine degradation I, and glycogen synthesis, whereas downregulated genes are involved in the immune system and in signal transduction. In a continuously cropped *C. tangshen* plant, the downregulated genes in leaves are psaA, psbA and the psbW [29]. The genes involved in various pathways (sucrose and starch metabolism, unsaturated fatty acid biosynthesis, phenylpropanoid biosynthesis, photosynthesis and plant hormone signal transduction, among others) are mainly regulated by soil amendments, which have a growing impact on plants in CC [102]. Different medicinal plant transcriptomics analyses with continuous cropping are shown below (Figure 2).

**Figure 3:**
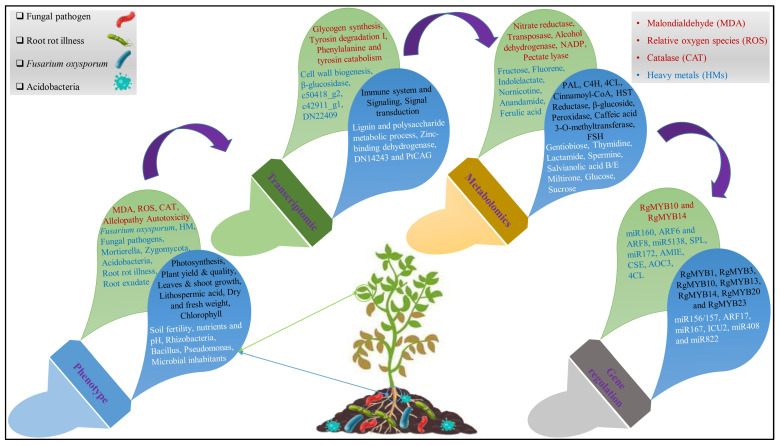
Overview of physiological, transcriptomic, metabolomics, and gene regulation networks of different medicinal plants in continuous cropping obstacles. The red and blue color in the green balloons indicates the upregulated genes in the aboveground and belowground respectively. Similarly, the black and white color in blue balloons indicates the downregulated genes in the aboveground and belowground respectively, in different medicinal plants.

Similarly, transcriptome analysis explains how the plant (*Pinellia ternata*) reacts to treatment with phenolic acids in CCO. The transcriptome showed that phenolic acid treatment elevated the DEGs associated with cell wall deterioration and ROS metabolism. Moreover, the levels of the critical metabolites were lowered due to downregulated DEGs in the pathways for phenylpropanoid production and metabolism of sucrose and starch (Figure 3). When combined, phenolic acids led to an overabundance of H_2_O_2_ and O_2_^−^, which led to the death of root cells, whereas *b-gentiobiose* and L-ascorbic acid efficiently reduced ROS stress [103]. In a plant transcriptome synthesis of *A. paniculata* for CC gene expression detection, 6193 uni-genes were substantially upregulated or downregulated, according to the expression of RNA-seq-based gene profiling. The most intricate genes in terpenoid biosynthesis, flavonoid biosynthesis, and phenylpropanoid biosynthesis in the *A. paniculata* plant were downregulated, showing that CC decreased the production of active ingredients by suppressing gene expression levels tangled in the biosynthesis pathways for these metabolites [104]. In addition, transcriptome investigation discovered that genes involved in energy metabolism and plant defense were suppressed, which decreased the patchouli plant’s ability to withstand CC stress [105]. The findings demonstrated that patchouli’s natural circadian cycle was disrupted by CCOs. Additionally, tryptophan biosynthesis, ubiquitin-mediated proteolysis, phenylalanine biosynthesis, sphingolipid metabolism, and tyrosine biosynthesis were often enriched throughout the CC period. Endocytosis, lacto, and neolacto series, glycosphingolipid production, folate biosynthesis, and endoplasmic reticulum protein formation were often shown to be considerably increased in CCO plants [106]. DEGs were mostly enriched in the abscisic acid-activated signaling pathway, plasma membrane, DNA binding transcription activity, reaction to chitin and the defense response, according to GO functional enrichment analysis. The genes involved in the metabolism of linoleic acid, cysteine, methionine, sucrose and starch metabolism, unsaturated fatty acid biosynthesis, and glycolysis/gluconeogenesis were upregulated. With p-HBA (p-hydroxybenzoic acid) therapy, genes related to plant–pathogen interaction, the MARK signaling pathway, and the signal transduction of plant hormone were downregulated. These pathways are connected to the rotting and browning of plant roots, both of which cause plant mortality [107,108]. Moreover, exogenous application of p-HBA stimulated the expression of genes associated with calcium-dependent protein kinase, dehydrogenase of ethanol, Ca^2+^/calmodulin-dependent EF-hand protein kinase, and pyruvate decarboxylase, and the expression of stunted genes associated with protein RPM1 disease resistance, peroxidase, and the lyase of phenylalanine ammonia [109]. Differentially expressed genes have been discovered via the analysis of transcriptome profiles of several plants under various conditions. Understanding the mechanism of CC and its effects on biological activities and metabolic pathways at the transcriptional level requires an understanding of the transcriptomic profile of different plants in response to crop stress. On these bases, CC may directly alter the transcriptome profiles of medicinal plants, and different genes in different pathways may show upregulated and downregulated responses in plants. A comprehensive (physiological, transcriptomics, metabolomics, and gene regulation (miRNA)) study of different medicinal plants in CCOs revealed different responses (Figure 3).

#### 3.2.2. Metabolomics Analysis

Metabolomics is employed in a wide range of studies to examine the adaptations and regulations occurring within complex systems, influenced by both internal and external factors [110]. In metabolomics research, MS (mass spectrometry) and nuclear magnetic resonance are the most often used detection methods. Due to benefits like high sensitivity and a broad dynamic linear range, MS is commonly employed [111]. CC ginseng soil was examined to inspect metabolomics and to verify its autotoxic activity. The effects of allelochemicals on ginseng’s development were used to screen and assess 23 ginsenosides and their contributions to autotoxic effects, which inhibit plant growth [112]. The metabolite profiles of several medicinal plants were altered significantly as a result of CCO. There is mounting evidence that during plant development, roots may release metabolites into the environment, and these metabolites can alter the characteristics of the soil and microbial community [113]. In CC, the levels of the majority of compounds, such as salvianolic acid B, dihydrotanshinone, rosmarinic acid, cryptotanshinone, miltirone, and tanshinone IIA, were markedly downregulated in *Salvia miltiorrhiza* Bunge (SMB) [114]. Polyamine levels (spermidine and spermine), directly associated with the development of SMB plants, were drastically downregulated in CC. The amounts of oligosaccharides necessary for single transduction of plant cells and the activation of the plant immune system were likewise much lower in SMB due to CC. The amount of salvinolic acid B/E was distinctly reduced in the SMB of continuously cropped plants [115].

Continuous cropping has been demonstrated in studies to alter plants’ metabolites and to decrease the amount of key therapeutic medicinal plant components [116]. Patchouli plant leaves’ metabolites such as cinnamic and carboxylic acids and their derivatives, prenol lipids, organooxygen compounds, and flavonoids changed significantly in response to CCOs [106]. Similarly, in a metabolomics study on *Panax quinquefolius*, 3, 4-dihydroxybenzoic, lignoceric, salicylic, palmitic, azelaic, heptadecanoic, cerotinic, benzoic, and oleic acid levels were significantly increased, while phytol, D-Talose, N-Acetyl-D-galactosamine, and mannose were significantly decreased [117]. This metabolites study not only supports our understanding of the metabolic profile of CCOs in medicinal plants, but also investigates its mechanisms in detail, which will be helpful in future metabolomics studies.

#### 3.2.3. Gene Regulation Network and miRNA Study

The challenges caused by CC are a complicated kind of stress that seriously impedes a viable increase in resources for pharmaceutical plants. The majority of plant life activities, including development and growth, metabolism, stress response, and hormone signaling are regulated by miRNAs [118]. Under different conditions, plants can increase the production of some miRNAs, and these can operate in targeted genes associated with stress, enabling them to adjust stress physiologically [119]. Overexpressing miR408 in transgenic chickpeas greatly improved drought stress tolerance in plants [120]. A *dh* rice mutant with overexpression of osa-miR171c demonstrated a substantial decrease in salt tolerance throughout the germination and seedling stages [50]. In response to alkali stress, the potato gene products sucrose-phosphate synthase (SPS) and shikimate O-hydroxycinnamoyl transferase (HCT) were negatively regulated by miR4243-X and novel m064-5p, respectively [121]. In recent years, both novel and conserved miRNAs have been discovered in medicinal plants due to rapid improvements in HTS (next-generation high-throughput sequencing) technology and analytical methods [122]. Worldwide, China has the largest diverse collection of medicinal plant germplasms; however, with the intensification of research efforts, the demand for these resources is also increasing [123]. Wild medicinal plants’ production is often low, and easily impacted by environments [124]. As a result, it is crucial to research the miRNAs involved in medicinal plants’ stress reactions. When comparing noncontiguous cropping and CC settings in *R. glutinosa*, miRNAs were probably responsible for continuous cropping challenges [125]. A total of 31 miRNAs from 14 different miRNA groups were found in *Salvia miltiorrhiza*, containing one particular miRNA that reacted due to CC [126]. According to the concept of sequence complementarity, miRNAs’ plant gene expression is controlled at the post-transcriptional level through two main mechanisms: translation suppression and targeted mRNA cleavage [127]. The function of miRNAs in gene networks controlling plant stress resistance may first be identified by elucidating the regulatory network of miRNA to mRNA that exists under distinct abiotic and biotic conditions [118].

Genomic studies in CC reveal the expressions of both mRNA and miRNA data of the root mechanism of the patchouli (*Pogostemon cablin*) plant [128]. Forty-seven miRNA-target gene pairings associated with defensive reactions, the development of roots, RNA synthesis, protein transport, signal transduction, and control of the flowering were discovered using combined mRNA-miRNA analysis. The stress of CC in patchouli plants activates the signal of calcium ions along with the MARK cascade, which in turn stimulates the downstream production of the number of early response genes. As a result of phosphorylation of the gene’s early response, which includes encoding receptor-like protein kinase and the threonine/serine protein kinase, normal gene expression and function are disrupted, leading to subpar plant development and even plant mortality. These genes also mediate metabolic disorders, the planned death of cells, and pathological phenomena. CC throughout this time altered the gene expression program involved in the plant’s normal development and growth, induced adjustments in major metabolic pathways, and triggered the production of several particular miRNAs linked with signal transduction and stress responses [28]. Different medicinal plants in a comprehensive study of continuous cropping obstacles, using transcriptomic, metabolomics, and gene regulation (miRNA), revealed different responses, as shown in Table 2.

### 3.3. Strategy to Deal with Continuous Cropping Obstacles

#### 3.3.1. Soil Amendments

Soil amendment is an effective substitute; chemical fumigation is an ecologically harmful alternative method for controlling soil-borne diseases, and also improves the soil’s physicochemical properties and the microbiota [11,134]. This, in turn, leads to enhanced constructive microorganisms for the control of plant disease, the destruction of pathogens (both fungal and bacterial), and the modulation of the immune system of plants [135]. Strawberry, apple, and prune replanting issues are effectively reduced by soil amendment [136,137]. An inexpensive and easily available resource that improves the health of soil and plants is cow dung (an organic soil amendment); this results in sustained agricultural production [138]. Apple replanting issues may be efficiently managed by combining biofumigants (*Brassica* species) with soil amendments [139]. Chloropicrin (CP) soil fumigation is a successful method to overcome CCOs and to enhance *P. notoginseng* plant quality, production, and N, P, and K upturns [140]. The addition of MOF (microbial organic fertilizer) may increase soil nutrients, and microbial diversity, alters the micro-ecological soil environment and modifies its purposes [141]. To satisfy the nutrition requirements of plants, fertilization is crucial in the development of *Citrullus lanatus* [142]. In *Arachis hypogaea* CC, after twenty years, organic fertilizer application increases the yield of the grains, reduces major diseases, including *Ralstonia* (bacterial wilt pathogen), and improves the rhizobacteria [143]. Bio-organic fertilizers can help with a variety of soil issues, including reductions in plant pathogens, soil-borne diseases, inhibition of watermelon *Fusarium wilt*, enhanced plant growth, resistance against stress, soil hydraulic and physical properties, microbial activities, root activity, SOD and POD activity, MDA contents, CAT, and plant photosynthesis [144,145]. In soil that has continuously grown soybeans for five years, chitin amendment affects the soil properties, microorganism community, and plant development [146]. Crude chitin and pure chitin both significantly raised the pH and nutrient availability of the soil, aided in plant growth, and enhanced the soil’s microbial activity. Ongoing cropping challenges in soybean plants could also be overcome with the help of soil-borne disease elimination [147,148].

The addition of helpful bacteria and fungi to the soil is one biotechnological management strategy that enables us to address the degradation of soil without harming the ecology of agricultural land [149]. AMF (arbuscular mycorrhizal fungus) occurs in over 80% of plant species; it is one of the most prevalent beneficial microbes, and enhances plant tolerance and resilience to abiotic and biotic stresses [150,151]. Soil nutritional availability increases due to the collaboration of AMF with other rhizosphere microbes. Some 10% of the bacteria in litter soil reacted to AMF *Glomus hoi* either favorably, like Firmicutes, or negatively, like Bacteroidetes and Actinobacteria [152]. In the rhizosphere of shrubs, AMF increased the richness of Gemmatimonadetes and Anaerolineaceae bacteria, while in the tomato rhizosphere, it increased the abundance of *Pseudomonas* and *Bacillus* (Figure 3), and also enhanced beneficial microorganisms linked to delivering N and P in nutrient-limited situations [153]. AMF may also activate a plant’s defense system against root rot, which increases significantly with CC in *P. notoginseng*, *P. ginseng*, and *P. heterophylla* plants [154,155]. However, the use of AMF bio fertilizer removed these barriers to CC by raising the rate of AMF inoculation, enlisting beneficial *Streptomyces*, *Bacillus*, and *Pseudarthrobacter* rhizosphere microorganisms and the fungus *M. elongate*, as well as suppressing harmful microbes like *Candidatus solibacter*, and fungal pathogen-associated bacteria such as *F. solani* and *F. oxysporum* [156,157].

#### 3.3.2. Crop Rotation

Crop rotation (CR), plant inter-cropping, choosing resistant cultivars, and biological management are only a few strategies for overcoming the drawbacks of CC [158,159]. CR is the most established and traditional agronomic technique for maintaining water and nutrient balance; avoiding disease; insect, pest, and weed control; and boosting crop production [160,161]. By preventing the reproduction and development of pathogens or breaking the disease cycle, improving the soil microecological environment, and either directly or indirectly producing inhibiting substances or specific antagonistic microorganisms, CR deters/decreases the infection of pathogens [162]. For soil health and plant growth, a variety of soil bacterial communities is crucial [163]. The microbial activity of soil is frequently utilized as an indicator of soil function. CR is a supremely cost-effective and eco-friendly way to address CCOs [164]. CR on unchanged plots of soil decreases demand for exterior responses, thereby improving soil microorganisms and yield, since different crops have unique nutritional requirements. This can potentially affect SOC (soil organic carbon) stability by enhancing soil physiochemical properties and changing the soil microenvironment [165]. This technique is commonly used to increase the production of maize and soybeans, and compared to continuous maize cultivation, crop rotation upturns maize production by 5−20%. Crop yield modifications are ultimately caused by changes to the soil microbial biomass [166]. This may reduce the number of weeds and insects while also increasing the nitrogen supply, which eventually boosts output [167]. Crop rotation in American ginseng (AG) enhanced the water contents, phenolic acids, cinnamic acid, vanillic acid (due to external stress), and the pH of the soil, as well as reducing the catalase, phosphatase, and sucrase activity in CCOs [168]. The importance of certain crops in the rehabilitation of soil used for CC has never been emphasized before, and selecting the proper CR is more efficient than prolonging the cycle period. *T. asperellum* was discovered as a kind of continuous crop obstacle opponent in *Panax notoginseng* [169]. Cucumbers should be rotated with other crops to boost output, reduce environmental damage from fertilization, and raise the relative abundance of *Ohtaekwangia* and *Flavobacterium* [170]. Making maximum use of soil nutrient niches is essential, along with optimizing CR choices and developing a healthy crop rotation system, as is lowering the number of pathogens, diseases, and insect pests in the soil.

#### 3.3.3. Intercropping

**Figure 4:**
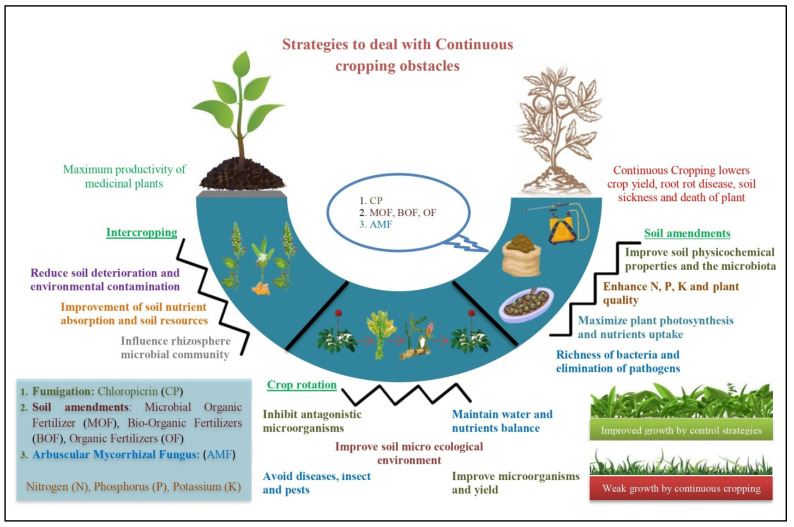
Different strategies to deal with continuous cropping obstacles in medicinal plants. Soil amendments; CP (chloropicrin), MOF (microbial organic fertilizer), BOF (bio-organic fertilizer), OF (organic fertilizer) and AMF (arbuscular mycorrhizal fungi) improve the plants’ growth and quality, and enhance the N (nitrogen), P (phosphorus), and K (potassium) contents. Crop rotation and intercropping also reduce the effects of continuous cropping.

Intercropping is the synchronized planting of two or more crops in the same field. In addition to facilitating the growth of medicinal plants, a rational intercropping system can increase yield and quality, while also increasing space utilization efficiency amongst species [171]. Therefore, intercropping has been proven to increase yield and plant development, and it can help with serious issues, including small crop production, buildup of pests and diseases, soil deterioration, and environmental contamination. Numerous investigations have demonstrated that the major benefits of intercropping include the reduction of pests and diseases, the improvement of the efficiency of soil resource use, and the improvement of soil nutrient absorption [172]. These benefits result from the interplay among crops and the alteration of microbial activity in the agricultural rhizosphere [173]. Intercropping has an impact on the structure, diversity, and functional diversity of the soil microbial community [174]. For instance, intercropping wheat with brassica altered the composition of the microbial population in the wheat rhizosphere [175], in a manner similar to how intercropping may influence the rhizosphere microbial community structure of maize and legumes [176]. Watermelon rhizosphere soil microbial communities became more diverse as a result of rice and watermelon intercropping, which in turn reduced the disease index of watermelon *Fusarium wilt* [177]. Intercropping peanuts with *A. lancea* decreased the accretion of autotoxic compounds in CC soil, improved the invertase and urease activity of the soil, inhibited the fungal population, and stimulated the bacterial community. In particular, the G-bacteria biomass intensely increased, and this was able to lessen the buildup of phenolic allelochemicals in the bulk soil and rhizosphere [178]. CCOs in medicinal plants can be controlled using different strategies, including soil amendments, crop rotation, and intercropping (Figure 4).

## 4. Conclusions

Obstacles to continuous cropping are now a significant factor impacting crop output and quality. Their negative effects include a decline in the fundamental chemical and physical characteristics of soil, modifications to microbial community structure, the buildup of autotoxins, weakened plant development, and an escalation of pests and diseases. In conclusion, CCOs in medicinal plants can be alleviated by using soil amendments to improve the microbial community and soil fertility. Transcriptomic and metabolomics studies provide insights into the molecular mechanisms involved in plant responses to CCO, which can guide the progress of new strategies to overcome these obstacles and improve medicinal plant production. Overall, a multi-pronged approach that incorporates soil amendments, crop rotation, and intercropping, along with a deeper understanding of plant–microbe relations and stress responses, may help us to overcome the obstacles associated with continuous cropping of medicinal plants.

Further study is required to recognize the most effective soil amendments and their application rates for different medicinal plant species and growing conditions. To mitigate these effects, farmers and researchers may need to implement strategies, such as crop rotation, intercropping, and soil amendments to maintain soil fertility and promote healthy plant growth, so that they can develop sustainable cropping practices to minimize these effects. By improving soil health, farmers can maintain high crop yields and ensure the viable production of medicinal plants.

## Figures and Tables

**Table 2:** Continuous cropping obstacles and transcriptomic, metabolomics and gene regulation study in different/medicinal plants.

Plant Name	Study Type	Upregulated Genes	Downregulated Genes	References
*Rehmannia glutinosa*	miRNA identification	miR160 (Auxin response), ARF6 and ARF8 (adventitious roots regulator), miR5138, SPL, miR172.	miR156/157, ARF17, miR167, ICU2 (flowering), miR408, miR822.	[125]
*Panax ginseng*	Transcriptomics analysis	Mitotic spindle elongation, enzyme inhibitor activity, centrosome cycle and duplication, carboxylesterase and pectinesterase activity, c41713_g1 (Invertase/pectin methylesterase inhibitor in leaves).	Photosynthesis, ion binding, cellulose metabolic process, polysaccharide and lignin metabolic process, C8930_g1 (Zinc binding dehydrogenase in roots), c41722_g1 (calcium-binding protein in the stem).	[99]
*Pogostemon cablin*	Transcriptomics analysis	AP2/ERF-ERF, bHLH, sesquiterpene synthase activity (GO: 0010334), exo alpha bergamotene biosynthesis (GO: 0045339), farnesyl diphosphate catabolic process (GO: 0045339), FAD binding (GO: 0071949).	MYB, HSF, bZIP, GARP-G2-Like, HB-HD-ZIP, water channel activity (go: 0015250), protein complex oligomerization (GO: 0051259), response to hydrogen peroxide (GO: 0042542), oligopeptide transport (GO: 0006857).	[28]
*Codonopsis tangshen*	Transcriptomics analysis	MYC2 (α-linolenic acid metabolism), EIN3 (ubiquitin mediated proteolysis), PR-1 (phenylalanine metabolism), CRE1 (zeatin biosynthesis), phenylalanine catabolism, tyrosine degradation I, glycogen synthesis, tyrosine catabolism, AP2, EREBP, WRKY, photosynthesis (PERK8, RAP2-4, DnaJ).	Immune system, signal transduction, mitotic activity, cell division, psaA (photosystem I), psbA (photosystem II), psbW (MSP), AHP (zeatin biosynthesis), GH3 (cell enlargement plant growth), CYCD3 (cell division), MYB, MADS, Jumonji family, trihelix, photosynthesis (BPS1, apoprotein, SR34A).	[29]
*Fragaria ananassa*	Transcriptomics analysis	Peroxidase activity (GO: 0004601), CNCGS, PR1 and WRKY transcription factors, cell wall (GO: 0005618), lignin catabolic process (GO: 0046274), heme and copper ion binding, response to virus (GO: 0009615).	FaWRKY33, nutrient transport and synthesis, glycogen phosphorylase activity, ammonium transmembrane transport (GO: 0072488), water transport (GO: 0006833), starch biosynthetic process (GO: 0019252).	[129]
*Salvia miltiorrhiza*	miRNA identification	miR156, smi-miR156a-1, miR396, miR319, pab-miR160a-like, smi-miR164a-1, miR166, leaf (miR031, miR021a, miR028), Stem (miR025a), miR165a-3p-like.	Root (miR031), NAC100-like (i5833_g1_i1), SPL13, athb-14-like (CL132Contig4), root growth ARF18-like, GRF3-like, athb-14-like.	[130]
*Beta vulgaris*	Metabolomics analysis	Terephthalic acid, 1,5-anhydroglucitol, fluorene, 3,4-dihydroxypyridine, anandamide, lactitol, salicylaldehyde, nornicotine, fructose, indolelactate, dihydroxyacetone.	Xylose, tyramine, gentiobiose, glucose, sucrose, lactamide, thymidine, neohesperidin, gentiobiose, pyridoxal phosphate, 5-alpha-dihydroprogesterone, cuminic alcohol, phytanic acid, valine.	[131]
*Andrographis paniculata*	Metabolomics analysis	Transposase, alcohol dehydrogenase (NADP+), nitrate reductase, pectate lyase, peptidylprolyl isomerase, NADP dependent sorbitol 6-phosphate dehydrogenase, β-glucoside gene, mevalonate 5-dinhophate decarboxylase (MVD, EC 4.1.1.33), isoflavonoid biosynthesis (1.1.4.1.3.21), apigenin (1.1.4.1.3.21), caffeic acid (6.2.1.12), Sinapyl alcohol (1.11.1.7), phenylalanine (4.3.1.24).	Flavonoids (Ko00941), terpenoids (Ko00900) and phenylpropanoid (Ko00940) biosynthesis, PAL, C4H, 4CL, Cinnamoyl-CoA reductase (CCR, EC 1.2.1.44), β-glucoside, HST gene (shikimate O-hydroxycinnamoyl transferase, EC 2.3.1.133), peroxidase (EC 1.11.1.7), caffeic acid 3-O-methyltransferase gene (COMT, EC 2.1.1.68), FSH gene, phosphomevalonate kinase (PMK, EC 2.7.4.2).	[104]
*Polygonatum odoratum*	miRNA identification	Nitrogen metabolism, phenylalanine, phenylpropanoid biosynthesis, tyrosine and tryptophan biosynthesis, phenylacetate synthesis (AOC3), AMIE, CSE, TYRAAT, 4CL, CYP98A, AOC3, ALDO, ASP5, HCT, PGD, SCRK, ADT, Ath-miR172a, novel_130, ath-miR172c, tcc-miR172d.	DNA replication, plant hormone signal transduction, brassinosteroid biosynthesis, syringyl lignin formation (EC: 1.11.1.7) CCR, CAD, and COMT, FBP, RPE, SORD, PGD, GLGC, GPI, E3.2.1.4, AROK, AROC, MAlZ, TYDC, GPD, E1.10.3.1, sbi-miR172f, osa-miR528-5p, mtr-miR2673a, mtr-miR2673a (EC: 1.11.1.7).	[132]
*Salvia miltiorrhiza*	Metabolomics analysis	Phenylalanine, ferulic acid.	Pehtasaccharide, Dihydrotanshinone I, spermine, salvianolic acid B/E, miltirone, spermidine, tanshinone II A, dehydromiltirone, tetrasaccharide, dehydrotanshinone IIA.	[115]
*Pinellia ternata*	Transcriptomic analysis	DN22409, DN59405, DN150689, DN14287, DN139642, 1,4-β-D-xylan synthase, DHAR, cell wall formation, β-glucosidase, APX, AOX.	PtCAG, PtSRK2, PtCCoAMT, phenylpropanoid biosynthesis, DN14243, DN11615, C4H, PtCSLD5, PtSS.	[103]
*Pogostemon cablin*	miRNA identification	pab-mir160c, ahy-mir408-3p, osa-mir397b, aof-mir398, novel20_master, mdm-mir1511, mdm-mir397a, stu-mir408a-3p, osa-mir397b, ahy-mir408–3p, stu-mir397–5p, aof-mir398, smo-mir408, mdm-mir397a, smi-mir12112, novel9_star, sly mir398a.	osa-miR397b, ahy-miR408–3p, mdm-miR397a, aof-miR398.	[106]
*Achyranthes bidentata*	Transcriptomic analysis	Gluconeogenesis (DLD, ENO, G6PDH, PGK, PGM, GPI, GALM, TPI, ADH1), glutathione metabolism (GR, LAP1, pepN, GGT3, SRM), plant–pathogen interaction (EIX1/2, RPM1, PR1, ETI, CAD, CERK1), signal transduction (SAUR, ARR, GID1, BSK, BZR1/2, CYCD3, CTR1, PR1, ARF, TIR1, AUX/IAA).	Glutathione metabolism (RRM2), gluconeogenesis (LDH, FBP, HK3, MINPP1, NAD+), plant-pathogen interaction (CALM, CDPK, WRKY33, BAK1, htpG, MKK4/5P, GsSRK), signal transduction (TGA, ERF1, ETR2, EIN3, EBF1/2, SNRK2, ABF, PP2C, BAK1, GH3), phenylpropanoid biosynthesis (PAL, C4H, 4CL, CCoAOMT, CAGT, HCT).	[133]

## Data Availability

Not applicable.
